# Can Phagocytosis, Neutrophil Extracellular Traps, and IFN-α Production in Systemic Lupus Erythematosus Be Simultaneously Modulated? A Pharmacological Perspective

**DOI:** 10.3390/ijms27020956

**Published:** 2026-01-18

**Authors:** Stephanie Seidlberger, Sindi Huti, Santos Castañeda, Michael Schirmer, Julian Fenkart, Georg Wietzorrek, Sandra Santos-Sierra

**Affiliations:** 1 Institute of Pharmacology, Medical University of Innsbruck, 6020 Innsbruck, Austriageorg.wietzorrek@i-med.ac.at (G.W.); 2 Rheumatology Division, Instituto de Investigación Sanitaria-Princesa (IIS-Princesa), University Hospital La Princesa, 28029 Madrid, Spain; 3 Department of Medicine, Universidad Autónoma de Madrid (UAM), 28029 Madrid, Spain; 4 Department of Internal Medicine II, Medical University of Innsbruck, 6020 Innsbruck, Austria; schirmer.michael@icloud.com

**Keywords:** SLE, therapeutics, TLR, phagocytosis, cytokines, NETs

## Abstract

Systemic lupus erythematosus (SLE) is an autoimmune disease with multiple and heterogeneous clinical manifestations (e.g., skin lesions, kidney damage, neuropsychiatric dysfunction), that primarily affects women and whose etiology remains unclear. Various therapies that regulate and reduce the immune system activity are in use or are being developed; however, many of them have serious side effects. Therefore, new approaches are needed to maximize remission periods and reduce associated side effects. In this review, we summarize the currently recommended therapeutic strategies. Furthermore, we hypothesize that the combined use of drugs targeting various dysregulated cellular processes in SLE (i.e., cytokine production, neutrophil extracellular traps (NETs), phagocytosis) might have therapeutic potential, at least in some disease phenotypes. Preliminary data show that Toll-like receptors 7/8 (TLR 7/8) inhibition (e.g., Enpatoran) may reduce interferon-α (IFN-α) production by monocytes and NET formation by neutrophils. Our hypothesis is that future therapies combining compounds that modulate the three cellular processes might result in a better disease management as current therapies.

## 1. Introduction

SLE is a chronic, autoimmune, and inflammatory systemic disease where the own immune system initiates an immune response against the host’s own derived epitopes. In this way, autoantibodies produced by the host’s B-cells orchestrate the formation of antigen–antibody immune complexes (ICs). Antigens may be of a different nature (proteins, polysaccharides, nucleic acids, lipoproteins, etc.) [1]. Characteristic autoantibodies found in SLE patients comprise the antinuclear antibodies (ANAs), anti-double-stranded DNA antibodies (anti-dsDNAs), anti-proliferating cell nuclear antigen (anti-PCNA), and anti-SM (autoantibodies against components of the small nuclear ribonucleoproteins—snRNPs). ICs may vary in size depending on factors like the valence of the epitopes (antigen determinants in a given molecule) or lattice (number of antigens and number of antibody molecules in a specific IC) [1]. These ICs are deposited or formed directly at the site of deposition in different tissues, where they activate the complement system and several types of receptors, leading to chronic inflammation and organ damage [2].

SLE manifests with both non-specific symptoms such as fatigue, fever, and weight changes and more specific manifestations, which vary from patient to patient, like skin lesions, painful and swollen joints (arthritis), muscle pain, hair loss, cell-blood counting alterations, or neuropsychiatric dysfunction (e.g., mood disorders, psychosis). One of the most severe manifestations of SLE involves kidney damage, known as lupus nephritis (LN). It presents with blood or protein in the urine and can lead to renal impairment or end-stage kidney failure. The symptoms differ significantly among individuals, ranging from mild to severe, and with alternate flares (periods of active illness) and remissions (periods with few symptoms/low disease activity) [3,4]. Cutaneous lupus erythematosus (CLE) comprehends a wide range of dermatologic manifestations which may or may not be associated with the development of a systemic disease. Depending on the severity, it is classified in several subtypes (i.e., acute-, subacute-, and chronic-cutaneous lupus erythematosus). CLE is two to three times more frequent than SLE [5].

The etiology of SLE is unknown, and due to the higher incidence in women between 15 and 44 years of age than in men (9:1), hormonal factors have been associated with disease development [6]. It is also speculated that certain environmental factors could trigger the disease in individuals with a genetic predisposition. Heritability is estimated at approximately 44%, and environmental factors and drug exposure (e.g., cigarette smoke, oral contraceptives, and hormone replacement therapy) seem to increase the risk of developing SLE. The role of additional factors such as ultraviolet (UV) light exposure, infections, pesticide contact, or obesity is insufficiently understood [7].

Certain medications are known to induce a lupus-like syndrome (drug-induced lupus, DIL). The symptoms can be similar to those presented by SLE patients, and while some antinuclear antibodies are present (e.g., against histones), dsDNA antibodies are not generally detected. In this case, the symptoms usually resolve upon discontinuation of the drug. Commonly associated drugs include antiarrhythmic agents (e.g., procainamide), antihypertensive agents (e.g., hydralazine, already listed out in several countries), antibiotics (e.g., minocycline), and anti-TNFα agents (etanercept, infliximab) [8].

In addition to the clinical presentation, the diagnosis of SLE is primarily based on laboratory findings with elevated erythrocyte sedimentation rate (ESR) and alpha-2/gamma globulins, decreased complement factors, hypochromic anemia, and normal C-reactive protein (CRP). The ANAs are generally elevated, and the anti-phospholipid antibodies may also be increased, pointing to a co-existing anti-phospholipid syndrome [9]. The diagnostic criteria follow the recommendation of the European Alliance of Associations for Rheumatology (EULAR)-2019 [10].

Patients are treated according to the disease severity (Table 1A). The mild forms of SLE, without visceral involvement, are treated with the disease-modifying antirheumatic drug (DMARD) hydroxychloroquine (HCQ; immunomodulator of TLR7 activity) and with nonsteroidal anti-inflammatory drugs (NSAIDs), to alleviate pain and inflammation. Glucocorticoids (GC; prednisolone) are also recommended, but for a limited period during inflammatory flare-ups. Alternatively, as a steroid-sparing strategy, immunosuppressant agents such as methotrexate (MTX), mycophenolate mofetil (MMF), azathioprine, cyclophosphamide, and tacrolimus can be used. The monoclonal antibody belimumab (inhibitor of B-cell activating factor) is approved as an adjunctive medication, although it is not appropriate for acute therapy as it has a latency of 3 months until healing activity is observed [11]. CLE patients are treated with appropriate topical and systemic agents (indicated in cases of widespread scarring, or treatment-refractory disease) such as GC and/or calcineurin inhibitors, and suitable education on sun protection [5].

In the cases of severe forms of SLE with organ involvement, the recommended treatment consists of high-dose pulses of prednisolone and/or immunosuppressants (MTX, azathioprine, cyclosporine A, or cyclophosphamide in the more severe cases). In addition, if the mentioned therapeutic options are unsuccessful, MMF or rituximab (anti-CD20 in B cells) can be considered. In treatment-refractory cases, CAR-T-cell therapy (with T cells modified to express receptors targeting CD19 on B cells, ending in B cell exhaustion) may also be implemented [12]. In parallel, treatment with antihypertensive drugs, lipid-lowering agents, anticoagulants, and bone-protective medications is recommended to avoid comorbidities [8].

The prognosis depends on the severity of organ involvement and response to treatment. Relapses and remissions are characteristic of the course of the disease. Nevertheless, earlier diagnosis and treatment have considerably improved survival rates (15-year survival rate of around 80%) [13]. Therefore, sustained remission and maintenance of low disease activity are crucial for reducing long-term complications and decreasing mortality.

Although the disease presents with heterogeneous and distinct clinical phenotypes, advancements in biomarker identification may help in defining cohorts of individuals at risk of developing SLE, likely helping in the response to treatment and relapse-risk predictions, which can potentially lead towards more personalized treatment strategies [14]. As mentioned previously, the 2019 EULAR/American College of Rheumatology (ACR) classification criteria of SLE patients include positive ANA, positive anti-dsDNA antibodies, and low C3 and C4 complement levels, highlighting the established role of some serological markers in the context of SLE [15]. The presence of other antibodies such as anti-phospholipid antibodies (anti-cardiolipin and anti-ß-2-glycoprotein) can be useful, although these antibodies are also positive in other immune diseases. For LN, IgE anti-dsDNA antibodies and low complement levels seem to be indicative of relapse [16]. Furthermore, anti-KIF20B antibodies have been identified as a potential biomarker for central nervous system disturbances associated with SLE (i.e., neuropsychiatric Lupus—NPSLE) [17].

## 2. Novel Approved Therapies Since the 2019 EULAR Guidelines

The latest drug approvals in SLE have been well documented in the recent literature [18,19] and they are shown in Table 1. It is worth mentioning the monoclonal antibody belimumab, which targets the B-cell activating factor (BAFF, or B-lymphocyte stimulator (Blys)), thereby inhibiting the survival of B cells (including autoreactive B cells) and thus reducing the production of autoantibodies. Already approved by the Food and Drug Administration (FDA) in March 2011, its efficacy in controlling disease activity has been supported by robust positive data in recent years [20]. Belimumab has also been approved for active LN, and a combination therapy with cyclophosphamide or MMF should be considered for this indication.

Additionally, a biologic that blocks IFN-α action by inhibiting the dimerization of the IFN-α receptor and its internalization (anifrolumab) received approval for extra-renal SLE in 2021 [21].

Finally, voclosporin is an oral calcineurin inhibitor (CNI), that blocks the activation of T-cells, and it has been approved in 2021 for patients with active LN in combination with background immunosuppression. In the case of active LN, add-on therapy with voclosporin (combined with MMF) should be considered [22].

**Table 1:** (**A**) Single treatments used in SLE. (**B**) Treatments combining drugs that affect different pathological processes in SLE. The studies referenced in the table are in addition to the EULAR 2019/2023 guidelines [15,23]. IV: intravenous. GI: gastrointestinal. SOC: standard of care. DAI: disease activity index. AE: adverse effects. BID: bis in die (twice daily).

(**A**)				
**Drug/Therapy**	**Indication**	**Mechanism of Action**	**Dosage/Application**	**Clinical Notes/Adverse Effects**
Hydroxychloroquine (HCQ)	All types of SLE, skin and joint involvement	TLR7/9 inhibition; cGAS activity inhibition [24]	Oral: 200–400 mg/day	Long-term therapy: risk of retinopathy, eye monitoring required
Glucocorticoids (GC)	Acute flares, crucial organ involvement	Genomic modulation: inhibition of inflammatory molecules transcription; rapid non-genomic pathway: blockage of phospholipase A2 activation, reduced lymphocyte activity, inhibition of ATP production [25]	Initial: 0.5–1 mg/kg/day orally for flares, then taper Maintenance: ≤2.5–5 mg/day IV pulse: Methylprednisolone 250–1000 mg/day × 1–3 days	Preferred short-term use; osteoporosis, diabetes, hypertension, glaucoma
Methotrexate (MTX)	Refractory skin/joint involvement, non-responsive to HCQ with or without GC, GC tapering not possible or higher dose of GC needed for skin involvement [26]	Di-hydropholate reductase inhibition; extracellular adenosine concentration increase [27]	Oral: 7.5–25 mg/week with 1–5 mg/day folate [27]	Bone marrow suppression, oral and gastrointestinal ulcers, alopecia, pulmonary toxicity, hepatic cytolysis [27]
Azathioprine (AZA)	Organ manifestations, non-responsive to HCQ with or without GC, GC tapering not possible, maintenance therapy in LN	Purine synthesis inhibition [28]	Oral: 2–3 mg/kg/day	Gastrointestinal disorders, mild infections, cytopenia [28]
Mycophenolate mofetil (MMF)	Skin and hematological manifestations, LN	Nucleic acid synthesis inhibition in T- and B-lymphocytes [28]	Oral: 2–3 g/day [29]	Leukopenia, diarrhea, nausea, infection risk [28,29]
Cyclophosphamide (CYC)	Severe LN, CNS involvement	DNA cross-link [28]	IV: 40–50 mg/kg over 2 to 5 days or 0.5–1 g/m^2^ monthly for 6 months (high-dose IV NIH regimen) [30] IV: 500 mg every 2 weeks for 3 months (EURO Lupus regimen) [31] Oral: 1.0–1.5 mg/kg/d (max 150 mg/d) for 2–6 months [31]	Infection risk, gonadal toxicity, neoplasms, amenorrhea [28]
Belimumab	Refractory SLE without severe organ involvement, additional to standard therapy in active LN	Anti-BLyS antibody [28]	SLE/LN: 10 mg/kg IV every 2 weeks (3 doses), then every 4 weeks SLE: 200 mg SC weekly LN: 400 mg SC weekly for 4 weeks, then 200 mg weekly [32]	Infection risk, hypersensitivity, nausea, headache, fatigue, psychiatric events [28]
Rituximab	Refractory severe cases, hematological involvement, CNS lupus, LN [33]	Anti-CD20 antibody [28]	IV: 375 mg/m^2^ weekly for 4 weeks or 1 g every 2 weeks (max 2 cycles/year) [28]	Off-label [34]; infusion reactions, infection risk [28]
Anifrolumab	Refractory severe cases with extensive skin disease involvement	IFNAR1/2 dimerization and internalization inhibition	IV: 300 mg every 4 weeks [35,36]	Infection risk, hypersensitivity, malignancy [37]
Calcineurin inhibitors (CNI; Tacrolimus, Cyclosporin, Voclosporin)	Skin disease, LN	NFAT dephosphorylation inhibition [28]	Tacrolimus: 0.1–0.3 mg/kg/day orally Cyclosporin: 2.5 mg/kg/day orally Voclosporin: 23.7 mg BID orally [38,39]	Nephrotoxicity, neurotoxicity, cardiometabolic complications, dermatological or GI complications [28,38]
(**B**)				
**Drug Combination**	**Dosage/Application**		**Efficacy**	**Adverse Effects**
MMF + Tacrolimus + Prednisone/Methylprednisolone	I. MMF 0.91 ± 0.12 g/day, TAC 3.65 ± 0.48 mg/day, Prednisone 35.7 ± 8.6 mg/day [40] II. MMF 1 g/day + TAC 4 mg/day + Methylprednisolone 10 mg/d [41] III. MMF 0.5–0.75 g/day + TAC 2–3 mg/day + Prednisone 10 mg/day [42]		Complete remission, decreased SLE-DAI, negative rate dsDNA and ANA, decreased proteinuria levels, increased serum albumin, normalized C3 [40,42] Increased tolerability; lower GI, leucopenia, irregular menstruation and upper respiratory infection incidence [40,42]	Increased new-onset hypertension, pneumonia, diarrhea [40,41,42]
Voclosporin + MMF + GC	Voclosporin 23.7 mg BID + MMF 2 g/d + GC (IV methylprednisolone on day 1–2, on day 3 oral prednisone 20–25 mg/d tapered to 2.5 mg/day by week 16) [43]		Complete remission, negative rate dsDNA [43,44]	GI and neurologic disorder, pneumonia and infection incidence; kidney dysfunction, hyperkalemia, diabetes and increase in blood pressure with CNI-class AE [43,45]
Belimumab + SOC (MMF or CYC-AZA)	Belimumab 10 mg/kg IV + SOC [46]		Complete remission, negative rate dsDNA [40,43,44]	Pneumonia and infection incidence [46]
Leflunomide + MMF/+ GC	Initial: Leflunomide 20 mg/day (3 months) + MMF 250 mg BID + prednisolone (0.8–1.0 mg/kg (6 weeks), 6–10 mg/day afterwards) Maintenance: Leflunomide 10 mg/day (6 months) + MMF 250 mg BID + prednisolone (0.8–1.0 mg/kg (6 weeks), 6–10 mg/day afterwards) [44]		Complete remission, negative rate dsDNA and ANA, decreased proteinuria levels Increased tolerability; lower GI, leucopoenia and irregular menstruation incidence [40,44]	Increased new-onset hypertension [40,44]

## 3. TLRs in Autoimmune Diseases and in SLE

The main role of TLRs is detecting the presence of invading pathogens in the host via recognition of pathogen-associated molecular patterns (PAMPs), coordinating the immediate response by innate immune cells. However, TLRs also recognize host-derived molecules from damaged or dying cells (damage-associated molecular patterns (DAMPs) or alarmins) initiating a sterile inflammatory process. In this case, the uncontrolled response to self-antigens (e.g., DNA/RNA, HMGB-1, S100 proteins, isoprenoid-derived DAMPs) [47] may become chronic and incite the activity of other immune cells (T- and B-cells). The over-activation of these may in turn trigger the emergence of autoimmune diseases like rheumatoid arthritis or SLE [48].

The initial hint on the involvement of TLRs in the SLE pathogenesis arose from animal studies. Primarily, studies in MRL/lpr mice treated with CpG (TLR9 ligand) or imiquimod (TLR7 ligand) indicated that activation of these two receptors led to more severe LN in comparison to controls. The effect seemed to be mediated through the induction of IL-6 production, which inhibits T-reg-mediated suppression of autoreactive T-cells [49]. Further, the lack of the TLR adaptor protein MyD88 (that is common to all TLR pathways except for TLR3) in knockout mice delayed mortality, prevented nephritis, and increased production of anti-dsDNA IgG, IFN-α, IL-12, IL-6, and IFN-γ. When the animals were treated with polyI:C (TLR3 ligand), they developed severe systemic autoimmunity, confirming the role of TLR3 in SLE. Furthermore, activation of TLRs can induce autoantibody production [50].

In humans, the role of TLR7 in the pathophysiology of SLE has been strengthened in recent years. TLR7 is activated by nucleic acid-containing ICs [51]. Additionally, other features underlie the role of this receptor in the inflammatory process in SLE: recently, a gain-of-function mutation (TLR7 Y264H) has been described [52]; an increased copy number of the TLR7 gene leads to higher protein expression [53] (and explains the higher female incidence observed); and single-nucleotide polymorphisms (SNPs) that stabilize TLR7 to degradation have been found in SLE [54]. The latest research highlights the role of platelet TLR7, which is essential for the formation of platelet-neutrophil complexes and low-density neutrophils (LDNs) in LN. This suggests that targeting platelet TLR7 could be a potential therapeutic avenue for this specific manifestation of SLE [55].

Due to the described TLR involvement in the pathophysiology of SLE, the development of TLR inhibitors, especially a dual inhibitor of TLR7 and TLR9, is being explored as a therapeutic strategy [56]. The double inhibitor has shown promising results in lupus-prone mice by reducing autoantibody production and ameliorating disease symptoms [57]. In addition, a phase I/II clinical study investigated E6742, a dual antagonist of TLR7 and TLR8, in patients with SLE. The study demonstrated an acceptable safety profile for E6742 and showed improvements in disease activity [58].

## 4. Investigational Therapies

Currently, new medicines targeting different hot spots in SLE are either under development or in an attempt to be adopted from other rheumatic conditions following their encouraging activity results. Upadacitinib is a second-generation selective Janus Kinase-1 (JAK1) inhibitor used in rheumatoid arthritis and psoriatic arthritis. In a phase II clinical trial, as monotherapy and in combination with Bruton’s tyrosine kinase (BTK) inhibitor, elsubrutinib reduced disease activity and flare frequency [59].

As previously mentioned, the dual TLR7/8 inhibitor E6742 showed a favourable safety profile and induced improvements in disease activity markers in phase I/II studies [58].

Following a different targeting approach, RSLV-132, a catalytically active human RNase fused to human IgG1-FC, has been designed to degrade extracellular RNA. A phase II trial showed potential benefits, however only in patients with systemic and high disease activity [60].

Other emerging potential therapies act by reducing macrophage activation and IC damage, like lysophosphatidic acid (LPA), trialled in animal models [61,62]; by blocking the receptor CXCR5 (with the antibody PF-06835375); or doubly antagonizing BAFF/APRIL (povetacicept) [63].

Also, CAR-T cell therapy with CD8^+^ CD19 CAR-T cells for depleting B-cells is being tested in patients with refractory lupus [12,64].

## 5. Defective Phagocytosis

Early on, it was shown that in SLE, dying cells (first apoptotic and then necrotic) are not properly removed from the organism. These cells release danger signals (e.g., DNA, RNA, nuclear proteins) that act as chronic immunogens for the induction of autoreactive lymphocytes and abrogation of B and T cell tolerance, and as antigens for IC formation [65,66,67].

In normal conditions, dying cells express ‘find me’ signals (e.g., released lysophosphatidylcholine or CX3CL1) and ‘eat-me’ signals (e.g., phosphatidylserine from the cell plasma membrane, which is exposed on the cell surface in apoptotic cells) enabling phagocytes to recognize and engulf them. Phosphatidylserine is either directly recognized by phagocyte receptors (e.g., Tyro-Axl-Mer (TAM) receptors, αvβ3 integrin, ß2-glycoprotein I receptor) or it binds serum proteins (e.g., β2-glycoprotein I, C1q and pentraxins), which facilitate their recognition [68]. Once apoptotic cells are phagocytosed, a plethora of anti-inflammatory cytokines (e.g., IL-10, TGF-β, PGE2, PAF) released by the phagocytes are responsible for controlling the inflammatory reaction [69].

In SLE, circulating monocytes and polymorphonuclear cells show a decreased engulfment of immunoglobulin opsonised beads when compared to healthy donors [70]. This can be due to a reduced expression of the cell adhesion receptor CD44 involved in the uptake of apoptotic cells [71]. Reduced C1q, C4, C3, or CRP, involved in the opsonisation of apoptotic cells [72], has also been observed in some individuals with severe SLE [73].

In addition to defects in apoptotic cell recognition, a deficiency in lysosomal maturation may result in impaired degradation of internalized apoptotic cell debris, as it has been described in macrophages from MRL/lpr mice. In turn, phagolysosomal membrane permeabilization may release dsDNA and IgG into the cytosol, leading to activation of cytosolic sensors and IFN-α production. Furthermore, intact apoptotic cell debris may also recycle back to the cell membrane and accumulate on the cell surface [74].

## 6. High Interferon Levels

IFN-α is persistently overexpressed in the majority of SLE patients, and it is a key immunopathogenic driver of SLE. It has been observed that therapeutic administration of IFN-α to patients for viral infections or tumours can induce autoantibodies and, occasionally, SLE. High serum IFN-α activity may also be a heritable risk factor in SLE. Initial studies in SLE patients described elevated serum levels of IFN-α, which correlated with both disease activity and severity. IFN-α levels also correlated with anti-dsDNA antibodies, complement activation, and circulating IL-10, and over time they have been associated with neuronal impairment in SLE [75]

Studies using type I IFN receptor knockout mice have shown beneficial effects on the spontaneous course of SLE, with mice lacking IFN-α/ß-receptors displaying significantly reduced autoimmunity, kidney disease, and mortality. Conversely, continuous in vivo expression of IFN-α dramatically accelerates the development of SLE manifestations in young lupus-prone mice [76].

The most active type I IFN (mostly IFN-α)-producing cells are plasmacytoid dendritic cells (pDCs). SLE-derived ICs containing nucleic acid from dying cells can induce IFN-α in pDCs, indicating their key role in SLE pathogenesis. pDCs express TLR1, 6, 7, 9, and 10, which might be involved in IFN-α production. Both exogenous and endogenous stimuli containing DNA or RNA could be potential TLR ligands [51]. The production of type I IFN by pDCs through TLR binding may induce maturation of myeloid DC (mDC), increase Th1 cytokine production (e.g., IFN-γ), promote Ig class switching in B cells, and increase IL-10 and Blys/BAFF in monocytes.

## 7. Neutrophil Extracellular Trap (NET) and Monocyte Extracellular Trap (MET) Production

Neutrophils mount an anti-pathogen response by releasing extracellular traps (NETs), web-like structures that are made of DNA, histones, and proteins (e.g., myeloperoxidase (MPO), neutrophil elastase). This process is called NETosis [77], and it may also be over-activated in sterile conditions, like in autoimmune diseases. In a normal setting, NETs are removed as part of the homeostasis recovery process, but in some individuals with SLE, predominantly those with LN, a deficiency in degrading NETs due to impaired DNAse-1 function can result in aberrant activation and self-damage [78].

NETs effects extend to different cell populations. They activate pDCs through TLR7, lower the T cell activation threshold, and stimulate DCs when these take up ICs activated by NETs. T and B cells turn autoreactive in the presence of NETs and produce autoantibodies [79,80]. Additionally, the interaction between platelets and low-density neutrophils (LDNs) is very important in the immunopathogenesis of SLE. High-density neutrophils require potent IFN-α signalling for NET formation, while LDN spontaneously forms NETs [55]. A link between LDNs, the neutrophil-to-platelet ratio (NPR), and the activity of SLE has been demonstrated, and thus it may serve as a predictive biomarker for flares of LN. In patients with active SLE, LDN levels are increased, with an immature phenotype particularly evident in individuals with kidney dysfunction. LDNs can preferentially bind to platelets, in a TLR7 expression-dependent way, leading to the formation of platelet-neutrophil complexes (PNCs) [55]. This interaction promotes NETosis, which can worsen the inflammation and cause tissue damage in SLE [81].

Some authors have demonstrated an abnormal functioning in the P-selectin Glycoprotein Ligand-1 (PSGL-1)/P-Selectin axis in a mouse model of systemic lupus and in patients with SLE. In fact, lack of P-selectin in mice induces the development of a lupus-like syndrome, and patients with cutaneous lupus have reduced P-selectin expression in skin vessels [82]. Recently, Muñoz-Callejas et al. observed that neutrophils from active SLE patients showed a reduced expression of PSGL-1 and low levels of PSGL-1 in neutrophils from SLE patients associated with the presence of anti-dsDNA antibodies, clinical lung involvement, and other SLE clinical manifestations [83]. In healthy donors, neutrophil interaction with immobilized P-selectin induced Syk activation, increased the NET percentage, and reduced the amount of DNA extruded in the NETs. In contrast, in active SLE patients, neutrophil interaction with P-selectin did not activate Syk or reduce the amount of DNA extruded in the NETs, which might contribute to increase the extracellular level of DNA and hence to disease pathogenesis [83].

In another study of the same group, monocytes from active SLE patients exhibited reduced levels of PSGL-1. Importantly, lower PSGL-1 levels in SLE monocytes are associated with several clinical manifestations, including anti-dsDNA autoantibodies, lupus anticoagulant, and anemia. However, in active SLE monocytes, PSGL-1/P-selectin interaction did not activate Syk or reduce the amount of extruded DNA. These data suggest a dysfunctional PSGL-1/P-selectin axis in active SLE monocytes, unable to reduce secondary necrosis or the amount of DNA released into the extracellular medium in monocyte apoptosis and DNA extrusion in extracellular traps (METs), potentially contributing to SLE pathogenesis [84].

## 8. Interplay Between Phagocytosis, NETosis, and IFN-α in SLE

The three cellular processes affected in SLE described above are closely linked, and defects in one of them may synergistically activate the pathological functioning of the others, perpetuating chronic inflammation and tissue injury in SLE patients. Defective phagocytosis leads to accumulation of apoptotic cell-debris and uncleared NET fragments. This activates neutrophils resulting in excessive NETosis. NETs, together with dsDNA, mtDNA, histones, LL-37, MPO, and autoantigens, induce the production of IFN-α by pDCs and monocytes [85]. In turn, IFN-α enhances NETosis, impairs phagocytosis by macrophages, and supports autoantibody production by B cells [86]. High circulating autoantibody levels drive the formation of ICs that again activate pDCs and complement, leading to a stronger IFN-α response [87]. Due to the functional connection of these processes, intervening in one of them might improve some aspects of the disease while simultaneously worsening other manifestations.

## 9. A Multi-Target Therapeutic Approach and New Potential Drugs

**Figure 1:**
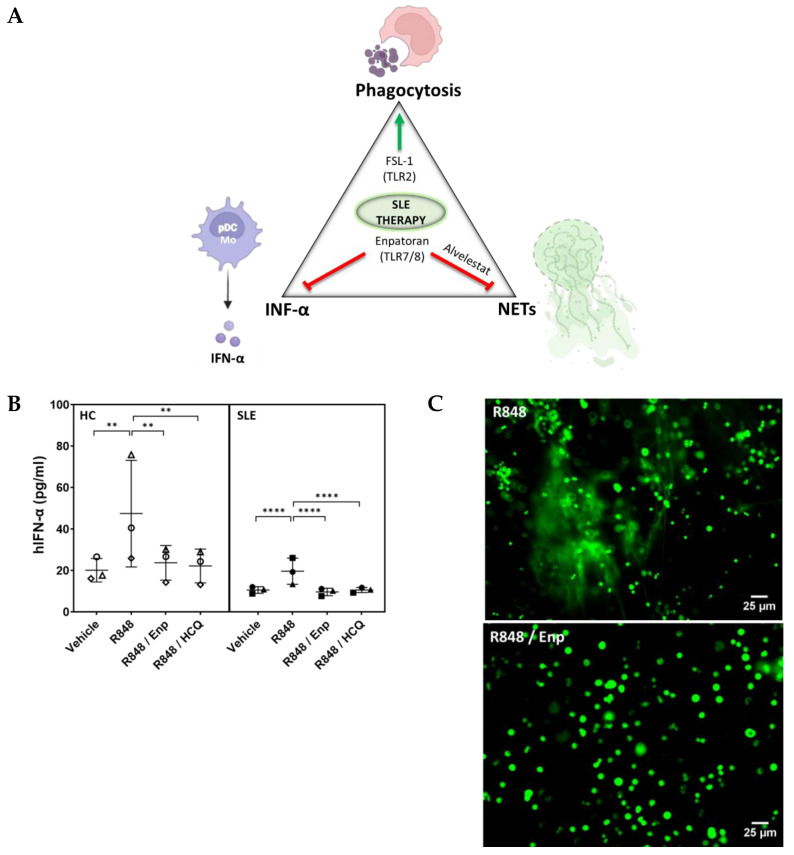
(**A**) Targeting three fundamental inflammatory processes in SLE might show beneficial therapeutic effect: (i) activating phagocytosis of dead cells via TLR low-level activation, for example, TLR2; (ii) decreasing IFN-α production via TLR7/8 inhibition; (iii) decreasing NET formation via elastase- or TLR7-inhibition. Green arrow: increase in activity. Red arrow: decrease in activity or inhibition. (**B**) Healthy-donor (N = 3) and SLE patients’ PBMCs (N = 3) were seeded in 96-well plates (1.5 × 10^5^ cells per well), and after overnight incubation, they were treated with Enpatoran (Enp; 4 µM; Invivogen, inh-m5049, San Diego, CA, USA), hydroxychloroquine (HCQ; 10 µg/mL; ThermoFisher, Waltham, MA, USA), or vehicle and they were stimulated with the TLR7/8 agonist R848 (1.6 µg/mL; Invivogen, tlrl-r848-10, San Diego, CA, USA). After overnight incubation, the IFN-α production was measured by ELISA (Invivogen, Leux-hifnav2, San Diego, CA, USA). Data represents the mean and standard deviation of three independent experiments. Each symbol denotes a different subject, either patient or control. Statistical analysis was performed using one-way ANOVA and Dunnett’s post hoc test. (**) *p* < 0.01, (****) *p* < 0.0001. (**C**) Healthy-donors’ neutrophils (N = 2) were seeded in 96-well plates (6 × 10^4^ cells per well) and they were treated with Enpatoran (Enp; 4 µM; Invivogen, inh-m5049, San Diego, CA, USA). NET formation was induced by TLR7/8 stimulation with R848 (2.5 µg/mL; Invivogen, tlrl-r848-10, San Diego, CA, USA). R848 led to NET extrusion, as demonstrated by DNA staining (SYBR^®^ Green I nucleic acid gel stain; Sigma-Aldrich, St. Louis, MO, USA) and acquisition of fluorescence microscopy images (excitation/emission: 480/520 nm). This effect was reduced by treatment with Enpatoran. Scale bars: 25 µm.

Because of the multisystemic nature of SLE, it is reasonable to think that a combination of therapies may show a better therapeutic forecast than single therapies, as it has already been implied for SLE (Table 1B) and other diseases [88]. Although the drug combinations used until now show superior efficacy to the current standard of care, they still present some adverse effects (e.g., MMF plus Tacrolimus plus GCs). We hypothesize that a combined modulation of different cellular processes underlying the SLE pathology might be therapeutically more specific with less adverse effects (e.g., reduction in IFN-α levels via TLR7 modulation, inhibition of NET production, and stimulation of phagocytosis (Figure 1A)). In addition, severe flares increase costs by about tenfold compared to mild flares, and thus, they should be minimized [89].

Targeting type I IFN activity, for example, with anti-IFN agents (e.g., rontalizumab, sifalimumab, S95021) or with an antibody–drug conjugate (BDCA2-ADC: litifilimab) [90,91,92], has shown promising results in SLE patients. However, treatment of SLE, particularly the severe variants, with biological agents can be expensive and can be associated with secondary adverse events. Additionally, the antibody administration must be performed by a healthcare professional, and the continuous therapeutic drug monitoring increases the costs [93]. Instead of using antibodies to block IFN-α activity, an alternative strategy is to target the main receptors involved in the cytokine production, that is, inhibiting TLR function, either by antagonizing the receptor or by inhibiting the activity of downstream proteins.

HCQ reduces IFN-α production via TLR7 inhibition [94] and, together with GC, remain the most valuable drugs in the treatment of SLE. However, HCQ can cause retinal toxicity, which can range from corneal deposits and retinopathy to irreversible damage of the retina [95,96], and patients treated with high-dose GC are subject to a plethora of complications [97]. Hence, additional and less toxic therapies are needed.

The use of new TLR7 and TLR8 antagonists to reduce IFN-α production has been trialled in preclinical studies in lupus mouse models, and some have entered early phases of clinical trials (e.g., Enpatoran-M5049 [98], Afimetoran-BMS-986256 [99], MHV370 [100], and E6742 [101]). In this respect, we have tested the Enpatoran inhibitory effect in SLE patients’ monocytes. Following TLR7/8-induced IFN-α production with R848, the decrease in cytokine levels by Enpatoran was comparable to the one produced by HCQ (Figure 1B).

In addition, there is experimental data on the reduction in TLR7 activity by inhibition of proteins that function downstream in the TLR pathway: Zimlovisertib (PF-06650833; IRAK4i) [102] was tested in preclinical studies; R835 (IRAK1/4i) [103] showed activity in human peripheral blood mononuclear cells (PBMCs), and a favourable safety in phase I studies; and Edecesertib (GS-5718; IRAK4i) [104] is currently in phase II clinical trials for cutaneous lupus erythematosus (NCT05629208) [105].

A second cellular activity affected in SLE that might be targeted is NETosis. The process of NET formation can be inhibited at different levels: targeting the enzyme responsible of histone citrullination peptidylarginine deiminase 4 enzyme (PAD4) with BMS-P5 or CI-amidine [106,107]; with an MPO inhibitor (IPF-1355) [108]; targeting the neutrophil elastase with AZD9668 (Alvelestat), which is investigated in the treatment of alpha-1 anti-trypsin deficiency [109]; with the JAK inhibitor Tofacitinib, which modulates NET formation and is under clinical trials in SLE patients (NCT02535689) [110,111]; or with the peptide inhibitor of complement C1 (PA-dPEG24), which has shown activity against NETosis [112].

In addition, HCQ (TLR7/9 inhibitor) and GC have shown efficacy in decreasing in vitro NET formation [113,114]. Here, as a proof of principle, we tested the effect of Enpatoran in NET formation by healthy controls’ neutrophils (Figure 1C). As it is appreciable, Enpatoran decreased the NETs induced by the TLR7/8 agonist R848; however, this result should be confirmed with SLE neutrophils, since differences to healthy control samples have been described (e.g., lower PSGL-1 in neutrophils [83]). In addition, factors present in the serum of SLE patients (e.g., medication, complement deficiency, ICs) may influence the response to experimental treatments.

Lastly, it has been shown that in phagocytes, TLR stimulation induces phagocytic activity to varying degrees via upregulation of genes involved in phagocytosis and dead-cell clearance [115]. Gardiquimod (TLR7/8 agonist) treatment of macrophages and DCs upregulates costimulatory molecules such as CD40, CD80, and CD86, which points to their enhanced activation [116]. Thus, it is not evident why SLE macrophages do not efficiently phagocytose the cellular debris produced in the course of the disease. To this point, it has been reported that TLR7/8 activation in neutrophils promotes cleavage of FcγRIIA on pDCs and monocytes, resulting in impaired clearance of ICs and increased complement C5a generation [117]. Possibly, phagocytosis instigation depends on the TLR that is activated. For instance, stimulation of the canonical TLR2 pathway leads to the production of cytokines like TNF-α but not IFN-α. Hence, agonism of TLR2 with minimal drug concentrations that would not lead to substantial TNF-α production might render improved phagocytosis without increasing IFN-α levels. However, the augmented phagocytosis strategy might not be effective in the case of patients with mutations in the complement system due to failure in opsonization activity [73,118].

## 10. Discussion and Future Directions

While the advantages of a therapy simultaneously targeting multiple pathological processes are clear (e.g., systemic global response, one-shot therapeutic intervention), several obstacles may arise from the pharmacological point of view: (i) drug-delivery issues (e.g., formulation and absorption incompatibility); (ii) pharmaco-kinetic/-dynamic challenges (e.g., additive, synergistic, or antagonistic effects); and (iii) toxicity issues (e.g., organ-specific damage and unpredictable reactions). Similarly, from the clinical perspective, management and monitoring of multiple medications and patients’ compliance are further challenges.

Moreover, the three proposed targeted activities are fundamental pillars of the host-immune response, and it is important to consider the adverse effects arising from a pan-inhibition. For instance, type I IFN has been shown to be protective in some mouse models of lupus [119], and a complete abrogation of IFN-α production might lead to a total immune suppression to viral infections, as was the case with the anti-IFN-α antibody sifalimumab (e.g., herpes zoster infections) [120,121]. Likely, inhibition of NET formation might be detrimental if exaggerated, increasing the susceptibility to infections and impairing pathogen clearance.

As shown before, inhibition of NET formation with Enpatoran is encouraging, as its safety profile in humans has been deemed adequate in Phase 1 [122,123] and in Phase 2 studies in CLE [124]. Enpatoran is orally bioavailable, and it was well tolerated by the study participants, without serious adverse episodes or death cases. Most adverse events were mild or moderate in severity (e.g., gastrointestinal disorders and infections). A steady state was achieved as early as day 3; the elimination half-life of Enpatoran after a single dose ranged from 7 to 12 h and was comparable between single and multiple dosing, allowing a twice daily dosing [122].

In addition, another possible candidate for NET inhibition, Alvelestat, is likewise orally bioavailable. In four clinical trials in participants with COPD and alpha-1 anti-trypsin deficiency, the most common adverse effects were headache, nausea, vomiting, and supraventricular extrasystoles. There were no clinically relevant changes in hematology, clinical chemistry, urinalysis, and vital signs. Following multiple doses, a steady state was reached in two days. The elimination half-life after repeated dosing was 5–15 h, which allows for twice daily dosing [125,126]. Accordingly, Enpatoran might be used in combination with Alvelestat or possibly other inhibitors like E6742 (IFN-α production) or HCQ to achieve a better inhibition of IFN-α and NET formation.

The use of TLR2 agonists like FSL-1, as drugs to activate phagocytosis, is limited due to its large molecular weight (1666.16 g/mol). Yet, some small molecules with TLR2-agonist effect have already been developed by our group [127] and other groups [128]. Up until now, only preclinical data is available on FSL-1 (e.g., mouse, non-human primates). The single subcutaneous administration of FSL-1 showed a 14.2 h half-life in non-human primates without long-lasting adverse clinical effects [129].

When testing combinations of the proposed compounds, the following interactions should be considered: (i) Since Enpatoran and Alvelestat show some gastrointestinal tract effects, a direct interaction by nausea/vomiting seems possible, thereby reducing each other’s bioavailability. (ii) Enpatoran and Alvelestat—although rarely—have both produced cardiac arrhythmias; since an additive effect cannot be excluded, ECG monitoring and exclusion of participants with a history of arrhythmias should be considered. (iii) Pharmacokinetics and pharmacodynamics of FSL-1 in humans are yet to be established in clinical trials.

## 11. Conclusions

In summary, we hypothesize that targeting three cornerstone processes associated with the pathogenesis of SLE (IFN-α production, NETosis, phagocytosis; Figure 1A), simultaneously, might show potential as an efficient therapeutic strategy once the different drug-combinations have been tested in a broader cohort of SLE samples.

## Data Availability

The original contributions presented in this study are included in the article. Further inquiries can be directed towards the corresponding author.
